# Knowledge and attitude of Saudi mothers towards their preschool children’s oral health

**DOI:** 10.12669/pjms.304.5069

**Published:** 2014

**Authors:** Ali M Al-Zahrani, Abdullah S Al-Mushayt, Meshari F Otaibi, Amjad H Wyne

**Affiliations:** 1 Ali M Al-Zahrani, MSc, Security Forces Health Center, Makkah Al-Mukarrama.; 2 Abdullah S Al-Mushayt, PhD, Pediatric Dentistry, Al-Qassim Private Dental College.; 3 Meshari F Otaibi, PhD, Security Forces Health Center, Makkah Al-Mukarrama.; 4 Amjad H Wyne, MDS, Department of Pediatric Dentistry and Orthodontics, King Saud University College of Dentistry, Riyadh. Saudi Arabia.

**Keywords:** Attitude, Knowledge, Mothers, Oral health, Preschoolers

## Abstract

***Objectives:*** To determine knowledge and attitude of Saudi mothers towards their preschool children’s oral health.

***Methods:*** One hundred and one mothers (of children aged 16 to 40 months) attending well-baby clinics at Security Forces Hospital Polyclinics in Makkah Al-Mukarrama participated in the study. A questionnaire was used to collect the required information.

***Results:*** A great majority (92.1%) of the mothers agreed that “baby teeth are important for child’s general health. Similarly, 90.1% of the mothers agreed that “using fluoridated toothpaste helps to prevent tooth decay”. About four in every ten mothers (43.6%) agreed that a child should be allowed to use a bottle at-will when he/she becomes able to hold it. More than half of the mothers (54.5%) agreed that letting baby sleep with bottle still in the mouth was of no harm to teeth. A significantly (*p*=0.04) higher percentage of high Socioeconomic status (SES) mothers as compared to middle SES mothers (85.9% versus 55.6%) agreed that “frequent feeding with milk or milk formula is of no harm to baby’s teeth”. A significantly (*p*=003) higher percentage of the middle SES mothers as compared to high SES mothers (66.7% versus 17.4%) agreed that a child should only visit a dentist in case of a dental pain/problem.

***Conclusions:*** The mothers need to be educated in several important areas related to feeding, diet and first dental check-up visit of their children.

## INTRODUCTION

Oral health is an integral component of preschool children’s health and well-being. Unfortunately, many children suffer from dental caries at an early age, even before they become 12 months of age.^1^ Those affected often have a reduced oral health-related quality of life as compared to their caries-free counterparts.^2^ Children with early childhood caries may also develop associated problems such as local infections, oral pain that also manifests as difficulty in eating and sleeping, reduced growth, psychosocial problems and increased risk of caries in permanent dentition.^2^^,^^3^ The primary dentition affected by dental caries at such a young age mostly has to be treated under sedation or general anesthesia, which carry its own risks.^3^^,^^4^

A number of risk factors have been cited in literature for early childhood caries that include; prolonged or at-will breast feeding, prolonged/frequent/nocturnal bottle feeding, family size or the child’s birth order, oral hygiene practices, dietary habits, and timing/reason for child’s first dental visit.^5^^,^^6^

Parents' oral health knowledge and attitude influence their children's oral health.^7^^,^^8^ The parents with appropriate oral health knowledge and attitude are likely to positively influence the oral health of their children.^8^^,^^9^ Mothers’ oral health knowledge and attitude in particular influence oral health of their children at an early age.^10^^,^^11^ However, very few studies have gauged the knowledge and attitude of mothers about their preschool children’s oral health.

 The aim of the present study was to determine knowledge and attitude of mothers towards oral health of their preschool children in Makkah Al-Mukarrama.

## METHODS

The study was conducted at pediatric clinics (well-baby clinics) of Security Forces Hospital in Makkah Al-Mukarrama. All the mothers fulfilling the following inclusion criteria participated in the study. The inclusion criteria included; no medical condition in their children and the child’s age between 16 to 40 months. A questionnaire modified from that used by Scroth et al (2007),^12^ was prepared in Arabic to collect information about mothers’ oral health knowledge and attitude towards oral health of their preschool children. 

The questionnaire was pre-tested and appropriate changes were incorporated to make all the statements comprehensible for the mothers. The mothers had the choice to agree or disagree with the statements. In addition to the demographic information such as number of children in family and socioeconomic status (SES) [monthly income}, the questionnaire included the following statements.

- Baby teeth are important for child’s general health

- Problems with baby teeth will affect child’s permanent teeth
- Decayed teeth can have effect on child’s general health
- Babies need their mouths cleaned even before eruption of teeth
- Using fluoridated toothpaste helps to prevent tooth decay
- Mother’s diet during pregnancy will affect baby’s teeth
- Good to always give baby a bottle to comfort while teething
- Frequently giving soft drinks is of no harm to child’s teeth
- Frequently giving juice is of no harm to child’s teeth
- Frequent feeding with milk or formula is of no harm to baby’s teeth
- Letting baby breast-feed all night is of no harm to baby’s teeth
- As baby gets older and can hold a bottle easily, he/she should use bottle whenever he/she wants
- Putting baby to bed with a bottle in mouth is of no harm to teeth
- Bottle feeding the child after 12 months of age is harmful for his/her teeth
- Breast feeding is important for the health of child’s teeth
- Babies who do not have bottles cry more
- Children should see a dentist latest by first birthday
- Children should see a dentist only when they have a dental problem

Ethical approval was obtained from the Research Ethics Committee of the Security Forces Medical Services. The purpose of the study was explained to all the participating mothers, and a written consent was also obtained. Anonymity of the information was ensured by removing all personal identifiers. The data were entered into a computer using SPSS Version #16; and frequency tables were generated. Chi-Square test was used to see any difference in mothers’ response in relation to number of children and socio-economic status. 

## RESULTS

A total of 101 mothers (of children aged 16-40 months) participated in the study. Thirty nine (38.6%) mothers were from middle SES and other 63 (61.4%) from high SES. The number of children in the family ranged from one to 16; majority of the mothers had five or more children (Table-I).

Mothers’ responses to various statements are listed in Table-II. A great majority (92.1%) of the mothers agreed that “baby teeth are important for child’s health”, and that “decayed teeth can have effect on the baby’s health” (96%). However, more than one-fourth (28.7%) of the mothers did not agree that “problems with baby teeth will affect child’s permanent teeth”. About nine in ten (88.1%) mothers agreed that “babies need their mouths cleaned even before the eruption of teeth”. A similar high percentage (90.1%) of the mothers agreed that “using fluoridated toothpaste helps to prevent tooth decay”.

Only 14.9% mothers disagreed with the statement that “it is good to always give baby a bottle to comfort while teething”. Similarly, only one-third (32.7%) of the mothers disagreed with the statement that “frequently giving soft drinks is of no harm to child’s teeth”. A similar trend was seen with use of milk or milk formula (16.8%) and breast-feeding all night (11.9%). The only exception was use of juices where 92.1% of the mothers disagreed with the statement that “frequently giving juice is of no harm to child’s teeth”.

More than four in ten mothers (43.6%) agreed that “when baby can hold a bottle easily, he/she should use bottle at-will”. More than half (54.5%) of the mothers agreed that “putting baby to bed with a bottle in mouth is of no harm to baby’s teeth”. In addition, only 44.6% of the mothers agreed that “bottle-feeding the child after 12 months of age is harmful for teeth”. Slightly more than half (52.5%) of the mothers agreed that “child should see a dentist latest by first birthday”; and one in every five (21.8%) mothers agreed that “children should see a dentist only when have a dental problem”. 

There was no significant difference in mothers’ response in relation to number of children in her family. However, two statements showed significant relation to SES of the mothers. A significantly (*p*=0.04) higher percentage (85.9.%) of high SES mothers as compared to 55.6.% of middle SES mothers agreed that frequent feeding with milk or milk formula is of no harm to baby’s teeth. On the other hand a significantly (*p*=003) higher percentage (66.7%) of the middle SES mothers as compared to 17.4% of the high SES mothers agreed that a child should only visit a dentist in case of a dental pain/problem

## DISCUSSION

A great majority of the mothers acknowledged the importance of deciduous teeth for the child’s general health, and that dental caries can affect their child’s health. This was encouraging in view of the fact that dental caries in deciduous teeth can affect children’s growth, result in significant pain, cause life-threatening infections, and diminish overall quality of life**.**^13^

**Table-I:** Number of children of the participating mothers

***Number of Children***	***Number***	***Percentage***	***Cumulative Percentage***
One	14	13.9	13.9
Two	29	28.7	42.6
Three	13	12.9	55.4
Four	15	14.9	70.3
Five or More	30	29.7	100
Total	101	100.0	

**Table-II:** Mothers’ response to statements regarding their toddlers’ oral health

***Statement ***	***Agree (%)***	***Disagree (%)***
Baby teeth are important for child’s health	93 (92.1)	8 (7.9)
Problems with baby teeth will affect child’s permanent teeth	72 (71.3)	29 (28.7)
Decayed teeth can have effect on child’s general health	97 (96.0)	4 (4.0)
Babies need their mouths cleaned even before eruption of teeth	89 (88.1)	12 (11.9)
Using fluoridated toothpaste helps to prevent tooth decay	91 (90.1)	10 (9.9)
Mother’s diet during pregnancy will affect baby’s teeth	86 (85.1)	15 (14.9)
Good to always give baby a bottle to comfort while teething	86 (85.1)	15 (14.9)
Frequently giving soft drinks is of no harm to child’s teeth	68 (67.3)	33 (32.7)
Frequently giving juice is of no harm to child’s teeth	8 (7.9)	93 (92.1)
Frequent feeding with milk or formula is of no harm to baby’s teeth	84 (83.2)	17 (16.8)
Letting baby breast-feed all night is of no harm to baby’s teeth	89 (88.1)	12 (11.9)
When baby can hold a bottle easily, he/she should use bottle at-will	44 (43.6)	57 (56.4)
Putting baby to bed with a bottle in mouth is of no harm to teeth	55 (54.5)	46 (45.5)
Bottle feeding the child after 12 months of age is harmful for teeth	45 (44.6)	56 (55.4)
Breast feeding is important for the health of child’s teeth	60 (59.4)	41 (40.6)
Babies who do not use bottles cry more	98 (97.0)	3 (3.0)
Children should see a dentist latest by first birthday	53 (52.5)	48 (47.5)
Children should see a dentist only when they have a dental problem	22 (21.8)	79 (78.2)

It was interesting to note that more than one-fourth of the mothers in this study did not agree that problems with deciduous teeth will affect permanent teeth. A direct relationship between caries in the deciduous teeth and increased likelihood of caries development in the permanent teeth has already been established since long.^14^ It is calamitous that this trend of separating the dental health of deciduous teeth and permanent teeth by parents still exists despite efforts by the dental profession to highlight the relationship between the two dentitions.

Nine in every ten mothers agreed that babies need their mouths cleaned even before the eruption of teeth. The American Academy of Pediatric Dentistry (AAPD) in its latest guidelines recommends implementation of oral hygiene measures no later than the time of eruption of the first primary tooth.^13^ Cleaning teeth as soon as they erupt helps in reducing bacterial colonization. Tooth-brushing should be performed for preschool children by a parent twice daily, using an age-appropriate size soft toothbrush. Flossing should be initiated when proximal contacts develop as proximal tooth surfaces cannot be cleaned with a toothbrush.^13^

A great majority of the mothers agreed that fluoridated toothpaste helps in preventing tooth decay. This was encouraging in view of the fact that potable water does not have optimal fluoride level in several areas of the Kingdom.^15^Optimal exposure to fluoride is important to all dentate infants and children.^13^ Use of fluoride for the prevention and control of caries is both safe and effective. In children considered at moderate or high caries risk under the age of two years, a ‘smear’ of fluoridated toothpaste should be used. In all children between two to five years, a ‘pea-size’ amount should be used. Professionally-applied topical fluoride, such as fluoride varnish, should be considered for children at high risk for caries. Systemically-administered fluoride should be considered for all children at caries risk who drink fluoride deficient water (<0.6 ppm) after determining all other dietary sources of fluoride. Careful monitoring is indicated in the use of fluoride-containing products.

Very few mothers in the present study disagreed with the statements that it is “good to always give baby a bottle to comfort while teething” and that “frequently giving soft drinks is of no harm to child’s teeth”. A similar trend was seen with the statements about use of milk/milk formula and breast-feeding. Improper feeding habits are important contributing factors for early childhood caries.^5^ Frequent consumption of liquids containing fermentable carbohydrates (milk, infant formula and soft drinks) increases the risk of caries due to prolonged contact between acidic/sweetened drinks and susceptible teeth.^16^^-^^18^ Parents are encouraged to have infants drink from a cup as they approach their first birthday, and wean children from the bottle at 12 to 14 months of age.^19^

Some results about the oral health knowledge of the mothers were disappointing. More than four in ten mothers agreed that a child should be allowed to use a bottle at-will when he/she becomes able to hold it. More than half of the mothers also agreed that letting the child sleep with bottle still in the mouth was of no harm to teeth. More high SES mothers as compared to middle SES mothers agreed that frequent feeding with milk or milk formula is of no harm to baby’s teeth. The opinion of high SES mothers about frequent feeding could be attributed to their higher educational, professional and social engagements; allowing them less time to comfort their child as compared to the middle SES mothers. A study of early childhood caries in Saudi preschool children has already proven nocturnal bottle feeding as a major caries risk factor.^18^ Studies in other parts of the world have also reported feeding at night and misuse of sugar to be the major contributors to the development of severe early childhood caries.^20^^-^^22^ Therefore, parents are encouraged to offer their infants milk and beverages in drinking cup by first year of life and stop bottle-feeding between 12 to 14 months**.**^19^

The AAPD supports the recommendations by the American Academy of Pediatrics regarding breastfeeding the child for at least one year. However, it mentions that frequent feeding at night including bottle-feeding and breastfeeding on demand are associated with early childhood caries.^13^The AAPD recommends that infants should not be put to bed with baby bottle and that breastfeeding at night should be avoided after the eruption of the first tooth.^13^ Night-time bottle feeding with juices, repeated use of a sippy or no-spill cup, and frequent in between meal consumption of sugar-containing snacks or sweetened/acidic drinks increase the risk of early childhood caries.^13^Previous studies in Saudi preschool children have already reported nocturnal bottle feeding, bottle feeding with sweetened milk and soft drinks, and at-will breast feeding as major risk factors for early childhood caries.^16^^-^^18^High-sugar dietary practices are established early by 12 months of age, and are maintained throughout early childhood.^13^ The American Academy of Pediatrics has recommended that children 1-6 years of age consume no more than 4-6 ounces of fruit juice per day from a drinking cup (not from a bottle or covered cup) as part of snacks or main meal.^13^

Though it was encouraging to note that more than half of the mothers agreed that child should see a dentist by his/her first birthday; yet, unfortunately one in five mothers also agreed that the child should see a dentist only when he/she has a dental problem. Furthermore, a significantly higher number of the middle SES mothers as compared to the high SES mothers agreed that a child should only visit a dentist in case of a dental pain/problem. This could be attributed to pressure on middle SES mothers to allocate financial resources to other matters. Wyne (2003)^23^ in his study of Saudi early childhood caries children has reported that the mean age of first dental visit in these children was much higher than the recommended age for first dental visit. The present recommendations for first dental visit range from as soon as the first teeth erupt to one year of age.^19^ It can be expected that an earlier routine visit to a dentist might prevent early childhood caries because it affords the dentist an opportunity to provide parents with information about healthy feeding habits and oral hygiene. The AAPD recommends that parents should establish a dental home for infants by 12 months of age.^19^ Providing anticipatory guidance regarding dental and oral development, fluoride status, non-nutritive sucking habits, teething, injury prevention, oral hygiene instruction, and the effects of diet on the dentition are also important components of the initial visit.^13^

The present study has gathered information on oral health knowledge and attitude of mothers of toddlers. Although the mothers’ answers were mostly appropriate, yet it became evident that immediate correction of knowledge and attitude was needed in several important areas. The results of the study will help dental fraternity in prevention of dental caries in preschool children.

## CONCLUSIONS

The mothers need to be educated in several important areas such as use of soft drinks and juices, giving baby-bottle containing milk/sweetened/acidic drinks before or during sleep and importance of early first dental check-up visit.
